# Dopaminergic Therapy Increases Go Timeouts in the Go/No-Go Task in Patients with Parkinson’s Disease

**DOI:** 10.3389/fnhum.2017.00642

**Published:** 2018-01-04

**Authors:** Xue Q. Yang, Brian Lauzon, Ken N. Seergobin, Penny A. MacDonald

**Affiliations:** ^1^MacDonald Lab, Brain and Mind Institute, University of Western Ontario, London, ON, Canada; ^2^Clinical Neurological Sciences, Schulich School of Medicine and Dentistry, University of Western Ontario, London, ON, Canada

**Keywords:** Parkinson’s disease, dopaminergic therapy, Go/No-go task, motor impulsivity, striatum

## Abstract

Parkinson’s disease (PD) is characterized by resting tremor, rigidity and bradykinesia. Dopaminergic medications such as L-dopa treat these motor symptoms, but can have complex effects on cognition. Impulse control is an essential cognitive function. Impulsivity is multifaceted in nature. Motor impulsivity involves the inability to withhold pre-potent, automatic, erroneous responses. In contrast, cognitive impulsivity refers to improper risk-reward assessment guiding behavior. Informed by our previous research, we anticipated that dopaminergic therapy would decrease motor impulsivity though it is well known to enhance cognitive impulsivity. We employed the Go/No-go paradigm to assess motor impulsivity in PD. Patients with PD were tested using a Go/No-go task on and off their normal dopaminergic medication. Participants completed cognitive, mood, and physiological measures. PD patients on medication had a significantly higher proportion of Go trial Timeouts (i.e., trials in which Go responses were not completed prior to a deadline of 750 ms) compared to off medication (*p* = 0.01). No significant ON-OFF differences were found for Go trial or No-go trial response times (RTs), or for number of No-go errors. We interpret that dopaminergic therapy induces a more conservative response set, reflected in Go trial Timeouts in PD patients. In this way, dopaminergic therapy *decreased* motor impulsivity in PD patients. This is in contrast to the widely recognized effects of dopaminergic therapy on cognitive impulsivity leading in some patients to impulse control disorders. Understanding the nuanced effects of dopaminergic treatment in PD on cognitive functions such as impulse control will clarify therapeutic decisions.

## Introduction

Parkinson’s disease (PD) is the neurodegenerative disease with the second highest prevalence rate, affecting approximately 1% of adults over 60 years of age in industrialized nations (de Lau and Breteler, 2006; Hirtz et al., 2007). Although symptoms of PD can occur throughout the lifetime, incidence rates of PD increase with age (de Lau et al., 2004; Pringsheim et al., 2014). The hallmark motor symptoms of PD include resting tremor, rigidity and bradykinesia (Jankovic, 2008).

In recent years, there has been tremendous interest in the autonomic and cognitive symptoms of PD (Poletti and Bonuccelli, 2013; Goldman and Postuma, 2014; Yang et al., 2016; Palmeri et al., 2017). Cognitive dysfunction in particular can cause devastating impairments to quality of life (Voon et al., 2009; Weintraub et al., 2015; Aarsland et al., 2017). The pathophysiological bases of cognitive dysfunction in PD are complex (Cools et al., 2001; Rowe et al., 2008; MacDonald and Monchi, 2011; MacDonald et al., 2011).

A central pathophysiological change in PD is significant degeneration of dopamine-producing neurons in the substantia nigra pars compacta (SNc; Dauer and Przedborski, 2003). The SNc is located in the midbrain, and primarily supplies dopamine to the dorsal striatum (DS) of the basal ganglia (DS; Dauer and Przedborski, 2003). The bulk of the caudate nuclei and putamina constitute the DS. In PD, dopamine depletion to the DS results in the cardinal motor symptoms (Dauer and Przedborski, 2003). In addition to motor functions, the DS has been linked to cognitive functions (MacDonald P. A. et al., 2014). DS has been linked to aspects of cognition such as motor planning (Jankowski et al., 2009), decision making (MacDonald et al., 2011), cognitive flexibility (Cools et al., 2003), and response inhibition–in particular resisting attentional capture by salient stimuli (Ali et al., 2009) or responding with pre-potent or habitual actions (Ali et al., 2009; MacDonald et al., 2011; Robertson et al., 2015). Overall, it seems the function of DS is to promote more deliberate and considered stimulus and action selections (Benke et al., 2003; Ali et al., 2009; Cameron et al., 2010; MacDonald et al., 2011; Mestres-Missé et al., 2012; Hiebert et al., 2014b, 2017; MacDonald A. A. et al., 2014; Robertson et al., 2015).

The ventral tegmental area (VTA) is located adjacent to the SNc. The VTA is another dopamine-producing area in the midbrain (Haber and Fudge, 1997). The VTA primarily supplies the ventral striatum (VS) of the basal ganglia with dopamine, as well as the limbic and prefrontal cortices (Haber and Fudge, 1997). The VS is composed of the nucleus accumbens and the most ventral aspects of the caudate nuclei and putamina. In contrast to SNc, VTA is generally spared in PD, especially during the early stages of disease (Kish et al., 1988; Rakshi et al., 1999). As a result, the cognitive, motivational, and affective functions mediated by VTA-innervated brain areas are relatively unaffected in PD (Kish et al., 1988; Rakshi et al., 1999).

The motor symptoms of PD are successfully managed with dopaminergic medication. The most common dopaminergic treatments are L-3,4-dihydroxyphenylalanine (L-dopa) and dopamine agonists (DAs; Dauer and Przedborski, 2003; Connolly and Lang, 2014). L-dopa is a precursor of dopamine that crosses the blood-brain barrier into the brain and is converted into dopamine, acting as an exogenous source of dopamine for PD patients (Lang and Lees, 2002). DAs act directly at the dopamine receptor level and upregulate post-synaptic receptor activity (Blandini and Armentero, 2014). Current clinical practices involve titrating dopaminergic medications to best address the motor symptoms that PD patients experience (Connolly and Lang, 2014), while aiming to avoid or minimize motor, cognitive, autonomic, or psychiatric side effects associated with dopaminergic therapies.

Although dopaminergic therapies are highly effective at improving motor function, they have differential and complex effects on cognitive functioning (Cools, 2006; MacDonald and Monchi, 2011). Some cognitive functions are improved by dopaminergic treatment whereas others are impaired (Cools et al., 2001; Rowe et al., 2008; MacDonald and Monchi, 2011; MacDonald et al., 2011; Ganjavi and MacDonald, 2015). In particular, dopaminergic therapy improves decision making, especially in the face of ambiguity, as well as selective and divided attention, and cognitive inhibition–processes that have all previously been attributed to the DS (Benke et al., 2003; Rieger et al., 2003; Cools et al., 2006; Thoma et al., 2008; Pine et al., 2009; MacDonald and Monchi, 2011). Learning is the function most often worsened by dopaminergic therapy (Cools et al., 2001, 2003; Zink et al., 2003; Jensen et al., 2007; Humphries and Prescott, 2010; Simões-Franklin et al., 2010; MacDonald and Monchi, 2011; Esslinger et al., 2013; MacDonald et al., 2013a,b; Vaillancourt et al., 2013; Hiebert et al., 2014a,b; Vo et al., 2014; Anderson et al., 2016). Impulse control disorders (ICDs) also arise with L-dopa but at a much higher rate with DAs in PD (Pontone et al., 2006; Weintraub et al., 2014). ICDs include serious behaviors such as pathological gambling, binge eating and hypersexuality (Pontone et al., 2006; Weintraub et al., 2014) that can greatly impact quality of life.

Impulsivity is a multifaceted construct. Antonelli et al. (2011) distinguish cognitive/motivational vs. motor/performance impulsivity. Cognitive impulsivity is defined as an increased propensity toward seeking rewards and enacting riskier decisions to gain reward, coupled with impoverished learning from feedback (Antonelli et al., 2011). ICDs purportedly develop and are maintained through dopamine-therapy-mediated impaired cognitive impulsivity. Motor impulsivity refers to difficulty holding back pre-potent and more automatic or habitual behaviors, as well as impairment in cancelling responses that have been planned or initiated (Antonelli et al., 2011). Motor impulsivity predisposes patients to falls (Wylie et al., 2012).

The Go/No-go paradigm is a task commonly used to assess response inhibition and the ability to cancel or override pre-potent response tendencies (Rubia et al., 2001; Hamidovic et al., 2008; Antonelli et al., 2014). In this way, the Go/No-go task provides a measure of motor impulsivity. The standard version of the task involves presenting two visual stimuli, a Go signal and a No-go signal. When confronted with a Go signal, participants are expected to make a keypress response as quickly as they can. Conversely, when they encounter a No-go signal, participants are instructed to refrain from making any keypress response. To enhance the potential for assessing motor impulsivity, the Go signal should appear at a much greater frequency than the No-go signal, establishing “Go” as the pre-potent response. For example, trials are often made up of 75% Go signals and 25% No-go signals. The Go Timeout rate is the percentage of Go trials on which participants fail to respond within a pre-set deadline. Another dependent measure is the percentage of trials on which participants respond erroneously with a keypress in the No-go condition, referred to as the No-go Error rate. In this way, more impulsive, less considered responding is exemplified by: (a) lower Go Timeout rate; and/or (b) higher No-go Error rate. In contrast, less impulsive and more considered responding is characterized by: (a) higher Go Timeout rate; and/or (b) lower No-go Error rate.

The Go/No-go paradigm has been employed to investigate impulsivity and response inhibition in PD. Most studies focused on differences between various subgroups of PD (Pessiglione et al., 2005; O’Callaghan et al., 2013; Cohen et al., 2014; Marzinzik et al., 2015; Peterson et al., 2015). Other studies compared PD performance to that of healthy, age-matched controls (Nakashima et al., 1993; Cooper et al., 1994; Franz and Miller, 2002; Dujardin et al., 2013). However, few studies have sought to understand the effect of dopaminergic therapy on motor impulsivity in PD, contrasting performance in the ON and OFF dopaminergic states (Farid et al., 2009; Antonelli et al., 2014; Herz et al., 2014). Those that have investigated the effect of dopaminergic therapy have include neuroimaging measures as primary output and did not establish whether or not differences in behavior occur on vs. off dopaminergic therapy in PD patients. That is, to this point, these studies using the Go/No-go task to investigate motor impulsivity in PD have mostly failed to reveal significant group (i.e., PD vs. Control or PD subgroup comparisons) or ON-OFF differences (Farid et al., 2009; Antonelli et al., 2014; Herz et al., 2014). Unfortunately, these null effects have a number of possible interpretations. Most studies included very low numbers of participants and potentially were underpowered to detect differences. Further, the Go/No-go procedures in these studies often featured task parameters that failed to clearly establish a pre-potent Go response or confounded their measure with increased memory and decision-making load, either by having low proportions of Go trials or multiple Go and No-go stimuli, respectively (Antonelli et al., 2014; Herz et al., 2014).

In the single occasion to our knowledge when ON-OFF differences have been observed, these effects are not interpreted with respect to the effects of dopaminergic therapy on motor impulsivity or the ability to withhold pre-potent responses. Geffe et al. (2016) tested a version of the Go/No-go task in PD patients on and off dopaminergic therapy though they included an implicit learning component to their study which was in fact the focus (Geffe et al., 2016). Geffe et al. (2016) variant of the Go/No-go task involved a conditioning phase during which participants were presented with a series of stimuli consisting of one of three non-target cues or a target stimulus, such that one cue consistently predicted subsequent target presentation in the following trial. In the Go block, participants were instructed to make a keypress in response to the target stimulus. In the No-go block, participants were required to make keypress responses to all non-target cues and inhibit the keypress response for target stimuli. In addition, they had a deconditioning phase during which no particular non-target cue predicted the target stimulus. Geffe et al. (2016) found that for the No-go condition, PD patients off medication and healthy controls showed increased errors in the deconditioning phase, which was interpreted as evidence of implicit learning in the conditioning period. However, this increase in error rate was not observed for PD patients when on medication, which they interpreted as an impairment in implicit learning with the addition of dopaminergic medication. Given that dopaminergic therapy is known to adversely impact association learning, this interpretation is highly plausible. These effects could also be interpreted as evidence that dopaminergic therapy reduces impulsive responding (i.e., lower No-go error rate ON relative to OFF dopaminergic therapy). This latter account was not articulated by the researchers but remains a possible reinterpretation. Overall, due to the many differences between the Go/No-go task used by Geffe et al. (2016) (i.e., conditioning and deconditioning phases, blocked design of Go and No-go trials, four stimuli of which one is the target stimulus), straightforward inferences regarding the effect of dopaminergic therapy on motor impulse control were precluded. This also makes direct comparisons of this task with the Go/No-go task implemented in the current study difficult. Consequently, to our knowledge, this represents the first study to implement a straightforward Go/No-go paradigm in which clear Go responses were biased, and in which the impact of dopaminergic therapy on motor impulsivity in PD patients was unambiguously tested.

Our goal in this study was to elucidate the effect of dopaminergic therapy on motor impulsivity in PD. Toward this end, we tested PD patients on and off dopaminergic medication with the Go/No-go paradigm. PD patients took their usual dopaminergic therapy as prescribed by their treating neurologist in the ON Session. For the OFF Session, PD patients refrained from their dopaminergic therapy for 16–20 h as detailed in the “Materials and Methods” section. To our knowledge, this represents the first study to implement a straightforward Go/No-go paradigm that clearly established the Go response as the pre-potent response, in which the impact of dopaminergic therapy in PD patients was directly tested with an ON-OFF design.

In a previous study, we showed that healthy young controls using the same Go/No-go task that was implemented in the current study, evidenced greater Go Timeouts when they were taking the DA pramipexole relative to placebo, revealing more considered and less impulsive responding in the DA condition (Yang et al., 2016). This finding occurred despite the fact that there were no differences in response times (RTs) between ON and OFF medication states, making our findings consistent with differences in motor impulsivity, not motor ability (Yang et al., 2016). These results further corroborate findings from a study by Hiebert et al. (2014a). Off dopaminergic medication, PD patients evidenced greater motor impulsivity in the form of exaggerated facilitation in the congruent condition of a modified location Stroop task relative to performance of unmedicated age-matched controls. When PD patients were tested on their usual dopaminergic therapy, their performance was normalized. These studies suggested that dopaminergic therapy actually *reduces* impulsive responding on tests of motor impulsivity, contrary to the common understanding that dopaminergic medications, DAs in particular, *promote* (cognitive) impulsivity in PD, leading to serious ICDs. These findings highlight the importance of: (a) understanding impulsivity as a multi-faceted concept rather than a unitary construct; and (b) fully clarifying effects of dopaminergic therapy across a variety of cognitive functions.

Based on this previous research, here, we hypothesized that PD patients would evidence more impulsive responding in the OFF state. We expected that dopaminergic therapy would increase motor impulse control, reducing the tendency to enact pre-potent responses reflexively and habitually, resulting in more considered and cautious responding. Again, impulsive responding was expected to be indexed by: (a) lower Go Timeout rate; and/or (b) higher No-go Error rate. In contrast, more cautious and considered responding would be expressed as: (a) higher Go Timeout rate; and/or (b) lower No-go Error rate, as described above.

## Materials and Methods

### Participants

Twenty-seven PD patients (16 males, mean age 67.81 ± 8.64 years) were recruited from the University of Western Ontario and Health Sciences North Hospital in Sudbury, Ontario. Participants were pre-screened for inclusion and exclusion criteria. All PD patients had been previously clinically diagnosed with PD by a licensed neurologist and met the UK Brain Bank criteria for a diagnosis of PD (Hughes et al., 1992). Participants were excluded for the following reasons: neurological disorders other than PD (e.g., stroke, seizures, dementia, mild cognitive impairment), psychiatric disorders other than mild-to-moderate depression [i.e., 29/63 > on Beck Depression Inventory (BDI; Beck et al., 1996)] or anxiety [i.e., 36/63 > on Beck Anxiety Inventory (BAI; Beck et al., 1988)], or history of alcoholism or drug abuse. Further, PD patients were excluded if they were not treated with dopaminergic therapy. Two patients were taking entacapone as an adjunct to L-dopa. One patient was taking both entacapone and amantadine as adjunctive therapies. One patient was taking DAs alone as primary therapy. The remaining patients were taking L-dopa as their primary therapy: either L-dopa alone (*N* = 15), or L-dopa in combination with DAs (*N* = 8). The data of participants who scored below 24 on the Montreal Cognitive Assessment (MoCA) were excluded from analyses. One PD patient was excluded for this reason. Finally, participants were excluded if their mean RTs or error rates in the Go or No-go conditions fell outside 2.5 standard deviations of the Group mean for that Medication Session (i.e., outliers). Four additional PD patients were excluded for having data that were deemed outliers. Analyses were completed with the data of the remaining 22 PD patients. This study was carried out in accordance with the recommendations of the Health Sciences Research Ethics Boards of the University of Western Ontario with written informed consent from all subjects. All subjects gave written informed consent in accordance with the Declaration of Helsinki (World Medical Association, 2013). The protocol was approved by the Health Sciences Research Ethics Boards of the University of Western Ontario.

### Apparatus

The Go/No-go task was conducted on a desktop computer (LG model 73821B-10) using the Windows 7 Professional operating system and a 22.0″ monitor (LG Flatron W2242TQ) running on a resolution of 1600 × 900 pixels. Participants were seated approximately 50 cm away from the screen and used a keyboard (Logitech K120) to record their responses.

### Procedures

All participants completed two testing sessions on consecutive days at the University of Western Ontario or Health Sciences North Hospital. For the OFF Session, PD patients were instructed to abstain from taking L-dopa/carbidopa and entacapone for 12–18 h before the start of the session, and dopamine agonists (e.g., pramipexole, ropinirole, pergolide) as well as amantadine, rasagiline, and selegiline for 16–20 h before the start of the session. For the ON Session, PD patients were instructed to take all dopaminergic medications for PD as prescribed by their treating neurologist. ON-OFF order was randomly assigned and counterbalanced. After the exclusion of five PD patients as previously described, twelve participants had an ON-OFF medication order and the remaining ten participants had an OFF-ON order. All participants were debriefed about the details of the study once they completed the second session. Participants were compensated for their time and participation.

#### Pre-task Assessments

Demographic and clinical data [i.e., age, sex, education, years of education, handedness, PD duration, Levodopa Equivalent Dose (LED)] were collected from all participants. PD duration refers to the number of years since a diagnosis of PD. LED is a calculation of the daily dose of dopaminergic therapy in units of L-dopa equivalents. Calculation of LED (mg) for each PD patient was based on the theoretical L-dopa equivalence (Wüllner et al., 2010; Hiebert et al., 2014a) as follows: L-dopa dose (mg) × 1 + L-dopa controlled release (mg) × 0.75 + L-dopa × 0.33 if taking entacapone + amantadine (mg) × 0.5 + bromocriptine (mg) × 10 + cabergoline (mg) × 50 + pergolide (mg) × 100 + pramipexole (mg) × 67 + rasagiline (mg) × 100 + ropinirole (mg) × 16.67 + selegiline (mg) × 10.22.

Heart rate (HR), systolic blood pressure (BP) and diastolic BP were measured using an automated BP monitor (Omron model BP785N) at the beginning and end of each testing session. Participants were also given a self-reported visual analog scale (VAS) at these two time-points to assess subjective alertness (Bond and Lader, 1974).

To assess baseline cognitive functioning, PD patients completed general cognitive assessments in the ON state. These general cognitive assessments and questionnaires were the American National Adult Reading Test (ANART), MoCA and Controlled Oral Word Association Test (COWAT). The ANART is a measure of verbal intelligence that has been adapted for use in North America (Grober and Sliwinski, 1991). The MoCA is a validated cognitive screening tool used to detect mild cognitive impairment (Nasreddine et al., 2005). The COWAT is used to assess verbal and category fluency (Ross et al., 2007). Participants also completed the Barratt Impulsiveness Scale (BIS), Sensation Seeking Scale (SSS), Questionnaire for Impulsive-Compulsive Disorders in PD—Rating Scale (QUIP-RS), and New Freezing of Gait (NFOG) questionnaire. The BIS and SSS are validated questionnaires estimating trait impulsiveness (Patton et al., 1995) and sensation-seeking (Zuckerman et al., 1978), respectively. The QUIP-RS is a valid and reliable measure of ICD symptom severity (Weintraub et al., 2012). The NFOG is a questionnaire used to assess freezing of gait in PD (Giladi et al., 2000).

Additionally, all participants completed the BDI, BAI and Starkstein Apathy Scale (SAS) in both sessions. The BDI, BAI and SAS are commonly used assessments of depression (Beck et al., 1996), anxiety (Beck et al., 1988), and apathy (Starkstein et al., 1992) in PD populations. Motor function was assessed on both testing days using the Motor Subscale of the Unified PD Rating Scale (UPDRS; Goetz et al., 2008).

#### Go No-Go Task

The Go/No-go paradigm is commonly used to assess motor impulsivity. The task consists of Go trials and No-go trials. On Go trials, participants were asked to respond by making a keypress as quickly as possible when the letter “X”, the visual Go signal, was presented. On No-go trials, participants were instructed to withhold keypress responses, when the letter “K”, the visual No-Go signal, was presented. On every trial, either the letter “X”, the Go signal, or the letter “K”, the No-Go signal, appeared in the center of the screen. Participants were instructed to press the spacebar for “X” and avoid pressing any keys for “K”. The visual stimuli were presented for a maximum of 750 ms, or until participants responded with a keypress. A blank screen was presented for a random duration between 400 and 800 ms during the inter-trial interval. The letter “X” was presented on 75% of trials, and the letter “K” was shown in the remaining 25% of trials, in a random order. This ratio of Go to No-go trials was intended to establish the Go keypress as the pre-potent response. Participants were instructed to make responses as quickly and accurately as possible. On each testing day, participants completed a total of 256 trials, organized into two blocks of 128 trials each, with 10 s breaks at the midpoint of each block and for a slightly longer break between the two blocks.

### Data Analysis

Physiological measures (i.e., HR, Systolic BP, Diastolic BP and VAS Alertness) were compared using 2 × 2 analysis of variances (ANOVAs), with Medication (ON vs. OFF) and Time (Pre-Task vs. Post-Task) as within-subject variables. Affective measures (i.e., mean BDI, BAI and SAS scores) and dependent measures derived from the Go/No-go task were compared between ON and OFF Medication states using paired-samples two-tailed *t*-tests. The dependent measures for the Go/No-go task were: (a) Go RT, comprising the mean RT for responses that occurred prior to the 750 ms deadline; (b) No-go RT, consisting of the mean RT for erroneous responses provided in the No-go condition; (c) Go Timeout Rate, reflecting the percentage of trials on which participants failed to respond prior to the 750 ms deadline; and (d) No-go Error Rate, denoting the percentage of trials on which participants erroneously made a keypress response in the No-go condition. RTs were calculated as the time in ms between the onset of the visual stimuli and the keypress responses. Data values for Go RTs were trimmed if they fell more than 2.5 standard deviations from the mean Go RTs in each medication state for each participant. The same process was used to trim No-go RT values. Lower Go Timeout rates and higher No-go Error rates were indicative of greater motor impulsivity whereas higher Go Timeout rates and lower No-go Error rates indexed less impulsive responding. Go RTs and No-go RTs were analyzed using non-parametric two-tailed Wilcoxon Signed Ranks Tests, and Go Timeout Rate and No-go Error Rate were analyzed using paired-sample two-tailed *t*-tests, with the Bonferroni correction for multiple comparisons. Analyses were performed using Excel (Version 2016), IBM SPSS Statistics (Version 21), and GraphPad Prism (Version 6). Data were considered significant if *p* < 0.05.

## Results

### Demographic, Baseline Screening Cognitive, Affective and Physiological Measures

Demographic and cognitive measures are presented for PD patients (Table 1). Full demographic data is included as supplementary material (Supplementary Table S1). All PD patients were within 2.5 standard deviations of the group mean for the NFOG, BIS, SSS, QUIP-RS ICD, QUIP-RS Total, MoCA, ANART, COWAT FAS and COWAT Animal. UPDRS scores were compared between ON and OFF medication states using a paired-samples two-tailed *t*-test. PD patients showed significantly higher UPDRS scores off dopaminergic medication compared to on, which was expected (*t*_(21)_ = 10.139, *p* < 0.001).

**Table 1:** Average demographic and cognitive measures for non-excluded Parkinson’s disease (PD) patients.

Variable	Value	SD
N	22	-
Age	66.77	9.15
Sex	11 males, 11 females	-
Education	15.18	4.11
Handedness	20 right, 2 left	-
PD duration	5.23	5.71
LED	626.59	276.31
UPDRS—ON	17.43	6.25
—OFF	21.82	6.06
	*t*_(21)_ = 10.139	*p* < 0.001
NFOG	7.86	7.52
BIS	59.23	8.99
SSS	11.59	4.74
QUIP-RS ICD	13.05	8.62
QUIP-RS Total	24.91	14.70
MoCA	27.32	1.73
ANART	122.72	6.46
COWAT FAS	15.71	5.22
COWAT Animal	21.23	6.93

*Values are presented as group means ± SD unless otherwise listed. All values are in units of the respective questionnaire or task scale. N: number of participants; Education (years): number of years of secondary and post-secondary education; PD duration (years): number of years since PD diagnosis; LED (mg): Levodopa Equivalent Dose; UPDRS: Motor Subscale Score of the Unified PD Rating Scale/56, listed for ON and OFF medication; NFOG: New Freezing of Gait Questionnaire/28; BIS: Barratt Impulsiveness Scale/120; SSS: Sensation-Seeking Scale/40; QUIP-RS ICD: Questionnaire for Impulsive-Compulsive Disorders in Parkinson’s disease Rating Scale—Impulse-Control Disorders/64; QUIP-RS Total: Questionnaire for Impulsive-Compulsive Disorders in Parkinson’s disease Rating Scale—Total score/112; MoCA: Montreal Cognitive Assessment/30; ANART: American National Adult Reading Test/135.6; COWAT FAS (number of words): Controlled Oral Word Association Test FAS Task; COWAT Animal (number of words): COWAT Animal Task. UPDRS scores were significantly higher OFF medication (p < 0.001)*.

**Figure 1:**
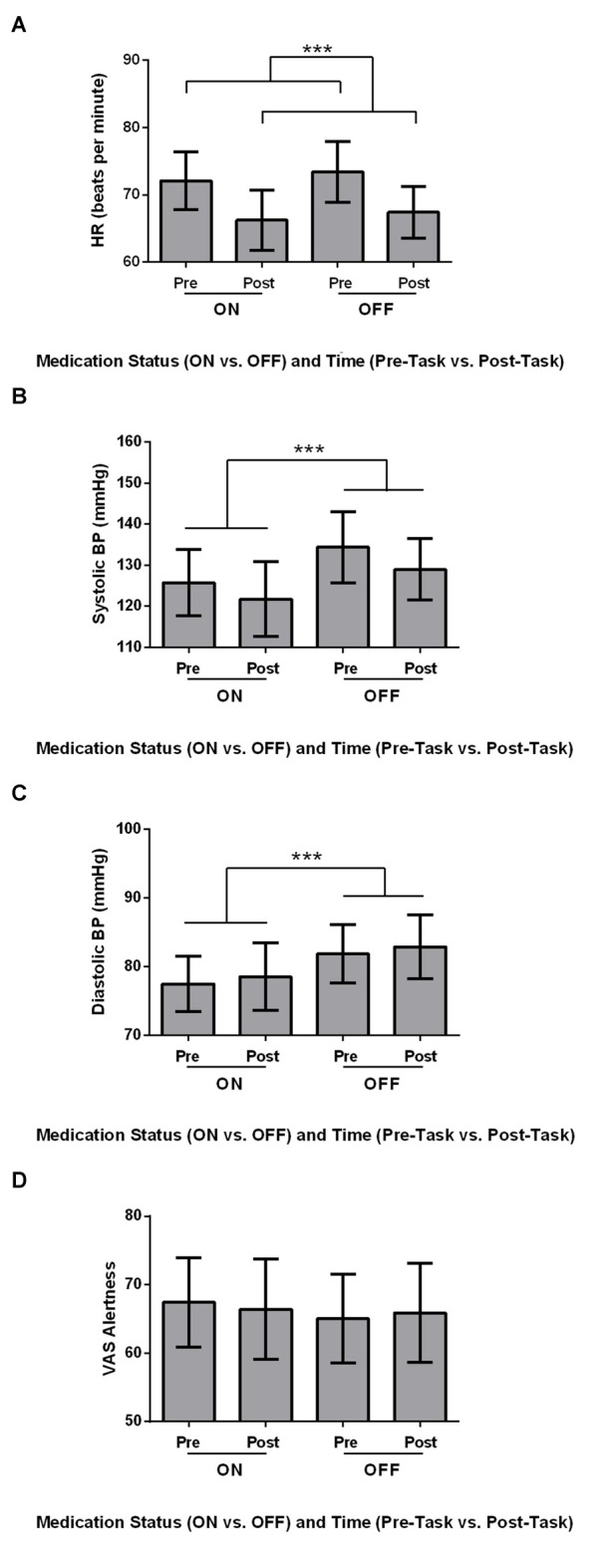
Physiological measures for Parkinson’s disease (PD) patients (*N* = 22). Values are presented as group means ± 95% confidence interval as per Masson and Loftus (2003). Data were analyzed using two-way analyses of variances (ANOVAs). **(A)** Heart rate (HR; beats per minute) was significantly higher Pre-Task compared to Post-Task (***p ≤ 0.001). **(B)** Systolic blood pressure (BP; mmHg) was significantly higher for the OFF Session compared to the ON Session (***). **(C)** PD patients had significantly higher diastolic BP (mmHg) OFF medication compared to ON (***). **(D)** No differences in visual analog scale (VAS) Alertness were found across Time and Medication (*p* > 0.05).

Physiological measures, including HR, Systolic BP, Diastolic BP and VAS Alertness were analyzed using 2 × 2 ANOVAs, with Medication (ON vs. OFF) and Time (Pre-Task vs. Post-Task) as within-subject variables. HR was significantly higher Pre-Task compared to Post-Task (Figure 1A; *F*_(1,21)_ = 24.569, MSe = 31.507, *p* ≤ 0.001). Additionally, Systolic BP was significantly higher OFF compared to ON (Figure 1B; *F*_(1,21)_ = 15.647, MSe = 88.459, *p* = 0.001). Diastolic BP showed a similar significant effect of Medication, with significantly higher Diastolic BP OFF compared to ON dopaminergic therapy for PD patients (Figure 1C; *F*_(1,21)_ = 11.743, MSe = 36.046, *p* = 0.003). For VAS Alertness (Figure 1D), no significant differences were found across Medication and Time (*p* > 0.05).

**Figure 2:**
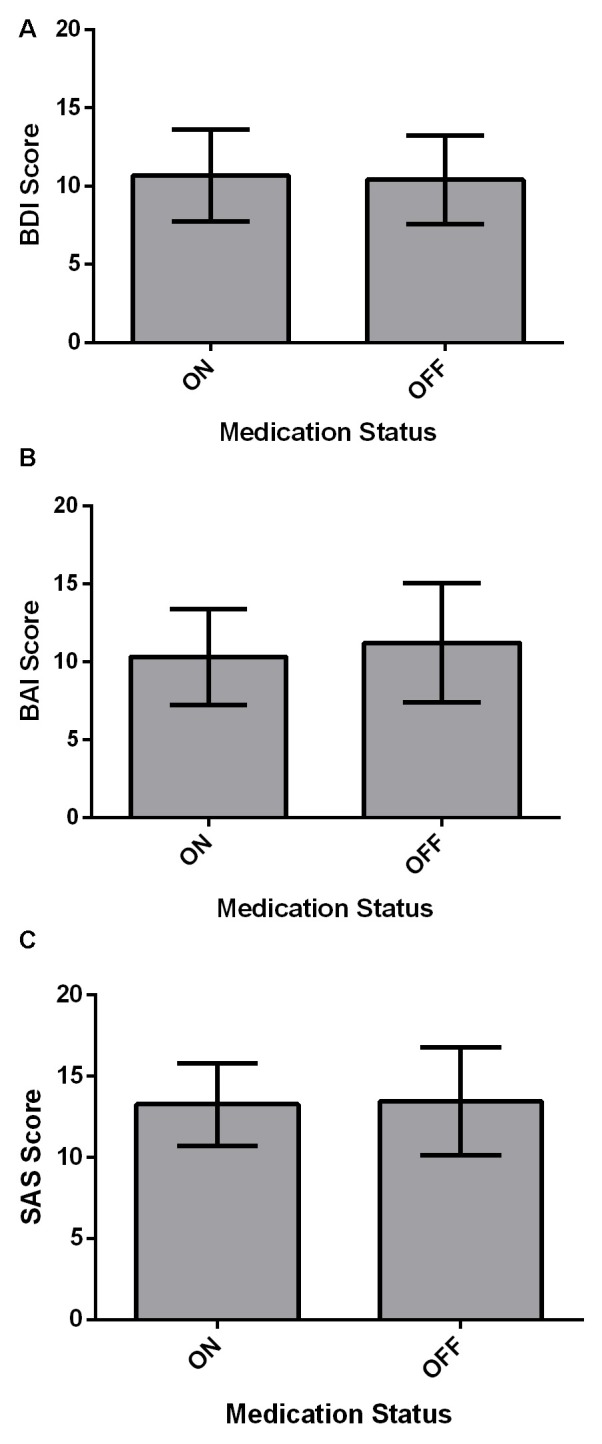
Affective measures for PD patients (*N* = 22). Values are presented as group means ± 95% confidence interval for repeated measures as per Masson and Loftus (2003). Affective measures were analyzed using paired-samples two-tailed *t*-tests. **(A)** PD patients did not significantly differ on the BDI between ON and OFF medication states (*p* > 0.05). **(B)** There was no significant effect of Medication state on the beck anxiety inventory (BAI; *p* > 0.05). **(C)** Starkstein Apathy Scale (SAS) score did not show a significant difference between ON and OFF states (*p* > 0.05).

Affective measures (BDI, BAI and SAS) were compared between ON and OFF medication states using paired-samples two-tailed *t*-tests (Figure 2). For all affective measures, there were no significant differences across Medication states (all *p* > 0.05).

### Go No-Go Task

**Figure 3:**
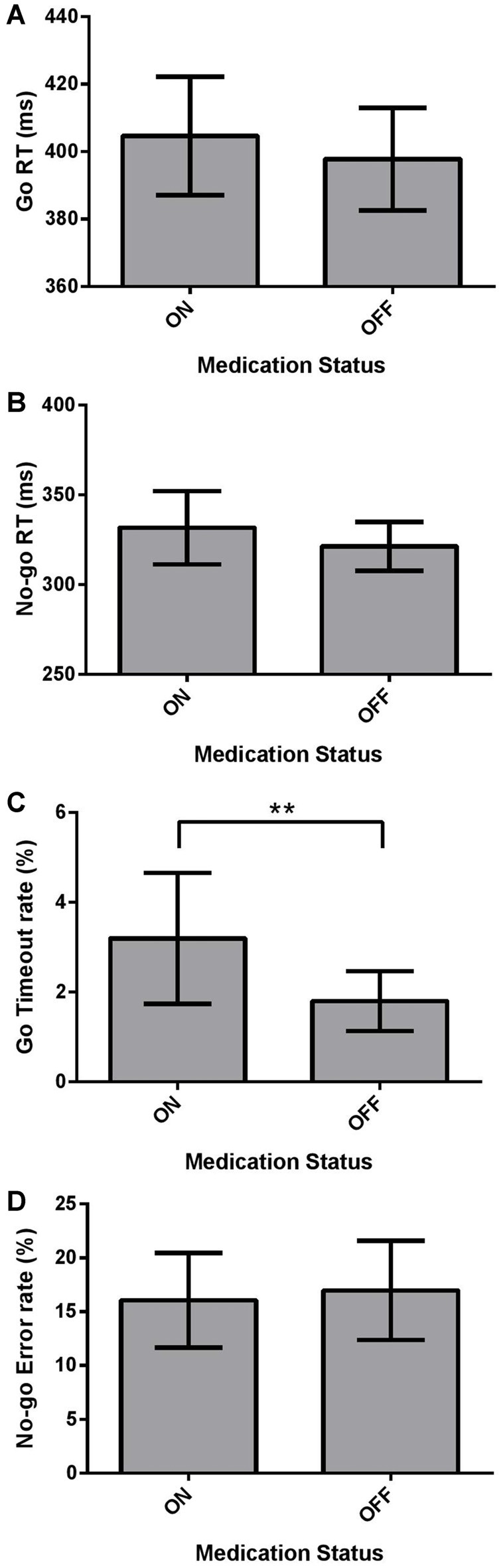
Dependent Go/No-go measures for PD patients (*N* = 22), ON and OFF dopaminergic medication. Values are presented as group means ± 95% confidence interval for repeated-measures as per Masson and Loftus (2003). Go Response times (RTs) and No-go RTs were analyzed using non-parametric two-tailed Wilcoxon Signed Ranks Tests, and Go Timeout Rate and No-go Error Rate were analyzed using paired-sample two-tailed *t*-tests, with the Bonferroni correction for multiple comparisons. **(A)** Mean Go RT was not significantly different for PD patients ON and OFF dopaminergic medication (*p* > 0.05). **(B)** No-go RT did not show a significant effect of Medication (*p* > 0.05). **(C)** PD patients had a significantly higher Go Timeout Rate ON dopaminergic medication compared to OFF (**p = 0.010). **(D)** No significant differences were found between ON and OFF Medication for No-go Error Rate.

We investigated the effect of Medication (ON vs. OFF) on the dependent measures of mean Go RT and No-go RT using two-tailed Wilcoxon Signed Ranks Tests, and Go Timeout Rate and No-go Error Rate using paired-samples two-tailed *t*-tests in the Go/No-go task using the Bonferroni correction. Mean Go RT was not significantly different for PD patients ON and OFF dopaminergic medication (Figure 3A; *p* > 0.05). No significant difference was found between ON and OFF No-go mean RT (Figure 3B; *p* > 0.05). PD patients ON had a significantly higher Go Timeout Rate compared to OFF dopaminergic therapy (Figure 3C; *t*_(21)_ = 2.851, *p* = 0.010) even after applying the Bonferonni correction (i.e., *α* = 0.0125). Examining No-go Error Rate, there were no significant effects of Medication (Figure 3D; *p* > 0.05).

## Discussion

We found that dopaminergic medication increased the Go Timeout rate in PD patients compared to their performance off medication. This suggests that dopaminergic therapy induced a more conservative response pattern for PD patients, *reducing* motor impulsivity in contradistinction to its widely-recognized *enhancement* of cognitive/motivational impulsivity producing ICDs in PD. We did not see a concomitant decrease in No-go errors for patients on relative to off dopaminergic treatment, however. In the No-go condition, a higher No-go Error rate in the ON state would also have signaled reduced motor impulsivity to parallel adoption of a more considered and conservative response strategy in the Go condition leading to more Go Timeouts. To engender a pre-potent Go response, there were far fewer No-go trials relative to Go trials. Consequently, it is possible that the No-go condition did not have the statistical power to reveal No-go error differences between ON and OFF medication states. Neither Go RT nor No-go RT were affected by medication status.

Comparisons of physiological measures showed that PD patients had lower HR post- relative to pre-Go/No-go Task. This trend of lower HR was fully expected because participants were sitting and inactive for the study period and had acclimatized to the novelty of the setting. PD patients also had increased systolic and diastolic BP off relative to on dopaminergic medication. This was anticipated as L-dopa is known to lower BP (Noack et al., 2014). Participants did not show any differences in subjective alertness, BDI score, BAI score, or SAS score across ON-OFF Sessions, demonstrating that our Go/No-go findings were not due to changes in alertness or mood between the two medication states.

By not presenting baseline PD performance relative to that of controls, we have not established abnormal control of motor responses (i.e., motor impulsivity) in the PD patients in our study. This was not our aim, though, as detailed below, reviews of this literature confirm that PD patients consistently exhibit deficits in inhibition of pre-potent responses and motor impulsivity (Kudlicka et al., 2011; Manza et al., 2017). Our objective was to explicitly investigate, in back-to-back tests within PD patients, the effect of dopaminergic therapy on motor impulse control using an accepted measure of this process (i.e., Go/No-go; Rubia et al., 2001; Hamidovic et al., 2008; Antonelli et al., 2014). Here, in PD patients, we entirely replicated the pattern that we observed in healthy young controls (Yang et al., 2016). Specifically, we previously showed that dopaminergic therapy increases the Go Timeout rate in healthy young controls. We previously interpreted this pattern of results, as we have here, as evidence that dopaminergic therapy increases control over motor responses and decreases the tendency to make more impulsive responses (Yang et al., 2016).

The alternative explanation that dopaminergic therapy simply slowed cognitive processes and/or motor execution rather than specifically promoting a more conservative response pattern is contradicted by other measures in our study, in addition to well-studied, established effects of dopaminergic therapy on behavior and the wider PD literature. Dopaminergic therapy did not affect overall RTs in our PD patients and it significantly speeded motor responses assessed with the UPDRS. Addressing bradykinesia and increasing the speed and fluency of movements and motor responses is the chief beneficial effect of dopaminergic therapy in PD (Espay et al., 2011; Macerollo et al., 2016). There is little evidence to suggest that dopaminergic therapy generally slows cognitive processes and in fact there is support that it hastens them (Cools et al., 2001; Shook et al., 2005; Hood et al., 2007; Righi et al., 2007; MacDonald and Monchi, 2011; MacDonald et al., 2011; Hanna-Pladdy et al., 2015). In contrast, as we review in sections below, dopaminergic therapy has been shown to increase response inhibition as well as to promote adopting a more conservative response criterion, consistent with our explanation for increased Go Timeouts in the ON-state for PD patients in our study.

It was also not possible for PD patients to be blinded to their medication status during the ON-OFF manipulation in our study. This is because patients had to comply with particular instructions to take or abstain from their usual dopaminergic therapy in a certain manner for ON and OFF session, respectively. Even if these instructions could be concealed, patients are well acquainted with their symptoms both on and off dopaminergic therapy which precluded blinding patients to our medication manipulation. Consequently, we cannot rule out the possibility that expectancy effects contributed to our results. However, as previously noted, dopaminergic medications are known to speed motor functions in PD patients (Espay et al., 2011) and consequently any expectancy effects would have acted contrarily to the results that we obtained. Overall, despite these acknowledged alternative interpretations, we interpret enhanced Go Timeout responses in the ON-state as evidence that dopaminergic therapy reduces motor impulsivity. This account for our findings is supported by a larger literature as detailed in the sections below.

### Effects of Dopaminergic Therapy on Go/No-Go Performance

There are few studies in the PD literature that have investigated motor impulsivity using the Go/No-go in PD patients. Fewer still have investigated the effect of dopaminergic therapy on performance though an important and concerning side effect of dopaminergic therapy is disordered impulse control. Herz et al. (2014) compared Go/No-go performance between PD patients with (*N* = 13) and without (*N* = 13) dyskinesia, and healthy controls (*N* = 13), with both patient groups being tested ON and OFF dopaminergic medication. Herz et al. (2014) used a variant of the Go/No-go task that included multiple Go responses (i.e., pressing either the left or right key) in addition to the No-go response. They did not find a modulation of Go/No-go performance by dopaminergic treatment. The added complexity related to multiple Go responses potentially reduced the pre-potency of Go relative to No-go, resulting in less difficulty withholding responses in the No-go condition. In another study, Farid et al. (2009) compared Go/No-go performance of PD patients (*N* = 9) ON and OFF medication relative to healthy controls (*N* = 9) who performed the task only once. They did not find behavioral differences between patients ON vs. OFF medication, or relative to performance of healthy older controls on Go/No-go accuracy or RT. However, with only nine participants in each group, the study likely was underpowered statistically to detect true differences if they occurred. Further, medication order was not counterbalanced. PD patients were always assessed in the OFF-ON order. In this way, and because healthy controls only performed the task once, order effects were confounded with medication effects. Antonelli et al. (2014) contrasted Go/No-go performance of PD patients (*N* = 7) ON and OFF the DA pramipexole. They found that administration of pramipexole increased impulsive choices on a delayed discounting task—their measure of cognitive impulsivity. However, no ON-OFF differences were observed on Go/No-go performance–their measure of motor impulsivity. This study was important in providing evidence that dopaminergic treatment affects distinct forms of impulsivity dissimilarly, supporting the idea that impulsivity is not a unitary concept, but rather is multifaceted. These results must be viewed with caution, however, considering that due to a sample size of only seven PD patients, this study was likely underpowered. Further, the authors’ rendition of the Go/No-go task involved presenting Go signals at 60%, and No-go signals at 40%, limiting the pre-potency of the Go response.

Geffe et al. (2016) used a variant of the Go/No-go task to assess implicit learning in de novo untreated PD patients OFF vs. ON a single dose of L-dopa. In the conditioning phase, a series of stimuli were presented such that one non-target prime stimulus acted as a reliable cue for presentation of the target stimulus in the subsequent trial. During the conditioning phase, participants learned to anticipate that the target stimulus would follow a particular non-target prime stimulus. Each conditioning phase was followed by a deconditioning phase, during which non-target stimuli and the target stimulus were presented randomly. PD patients off medication and healthy controls were found to make more errors in the No-go condition of the deconditioning phase. This was interpreted as evidence that associations between the prime stimulus and the target stimulus had been learned in the conditioning phase. This learning enhanced the anticipation that the target stimulus would follow, leading to more No-go responses. When PD patients were on medication, they evidenced less No-go errors in the deconditioning phase. The authors interpreted this as evidence that association learning between prime stimuli and target stimuli had been less well learned by patients treated with dopaminergic therapy. This finding is consistent with previous research showing that dopaminergic therapy impairs learning (Swainson et al., 2000; Cools et al., 2001, 2007; MacDonald and Monchi, 2011; MacDonald et al., 2011; Vaillancourt et al., 2013; Vo et al., 2014, 2017; Gallant et al., 2016). However, the fact that PD patients performed fewer No-go responses on dopaminergic therapy in the deconditioning phase could also be reflective of enhanced motor control. Due to the design, either interpretation is possible. There were substantial differences in task parameters and research goals between Geffe et al. (2016) study and ours. However, their results are not at odds with our findings.

In summary, we provide the first demonstration that dopaminergic therapy affects performance in a straightforward Go/No-go task in PD patients. The limited number of previous studies investigating the effects of dopaminergic therapy on Go/No-go performance in PD had small sample sizes and were impacted by other methodological issues, outlined above, that might reduce sensitivity to detect medication effects, predisposing them to null findings. We sought to redress these concerns by testing a sufficiently large number of PD patients (i.e., 27 PD patients) and setting the Go/No-go parameters at 75% Go trials, with a single Go stimulus, relative to 25% No-go trials, establishing a strong pre-potent Go response. We suspect that these factors explain discrepancies between our findings and the results of previous investigations of dopaminergic therapy on motor impulsivity in the Go/No-go task in PD patients.

Our finding of increased Go Timeouts for PD patients when tested ON relative to OFF dopaminergic therapy is entirely in keeping with our previous investigations of pramipexole vs. placebo on Go/No-go performance in young, healthy controls (Yang et al., 2016). Using the identical paradigm employed in the present study, we found that pramipexole produced increased Go Timeouts relative to performance on placebo. We interpreted this pattern as reflecting a more conservative response set owing to pramipexole (Yang et al., 2016). Entirely in line with our findings here, we also found no pramipexole-placebo differences in terms of No-go errors. As in the current study, there were far fewer No-go trials and hence we speculated that we were somewhat underpowered to detect medication-related effects in this condition.

### Effects of Dopaminergic Therapy on Cognition Including Impulsivity

Consistent with the notion advanced here that dopaminergic treatment in fact *increases* motor impulse control, Hiebert et al. (2014a) found that PD patients evidenced greater facilitation in the congruent condition of a modified Stroop task when tested OFF dopaminergic therapy relative to the degree of facilitation observed in healthy age-matched controls. Facilitation was normalized when PD patients were tested ON their usual dopaminergic therapy. We surmised that enhanced facilitation in the OFF state arose due to more impulsive and less considered responding, which was rectified by usual dopaminergic therapy. The current study and those presented above highlight the fact that dopaminergic treatment has varied effects on different aspects of impulsivity. These studies present evidence that dopaminergic therapy *reduces* motor impulsivity in contrast to the more widely-understood effect of *increasing* cognitive/motivational impulsivity producing ICDs in PD patients. This understanding is important for the clinical approach to PD and decisions regarding titration of dopaminergic therapy considering motor as well as cognitive symptoms.

Our observations in this study are in accordance with previous research on response inhibition and/or response withholding in PD generally. In a meta-analysis of the effects of dopaminergic medication and PD disease duration on measures of response inhibition, Manza et al. (2017) found that for studies of response inhibition with PD participants on dopaminergic medication, response inhibition deficits were significantly correlated with disease duration. The authors examined studies of common measures of response inhibition, including the anti-saccade, stop-signal, Stroop, and Go/No-go tasks. PD patients were found to have poorer response inhibition compared to matched healthy controls, in agreement with conclusions from another previous meta-analysis (Kudlicka et al., 2011). For studies with PD patients at earlier disease stages (i.e., <7 years since diagnosis), dopaminergic medication tended to improve the ability to inhibit inappropriate responses, resulting in performance that was worse than but approached the level of healthy controls (Manza et al., 2017). Conversely, studies with PD patients at later disease stages (i.e., >7 years since diagnosis) tended to find that dopaminergic medication worsened response inhibition compared to the unmedicated state. The current study investigated PD patients with an average disease duration of approximately 5 years (range 1–26 years), comparable to the patient samples in the studies examined by Manza et al. (2017) in their meta-analysis of PD patients at earlier disease stages. Our finding that dopaminergic therapy caused PD patients to enact more cautious responding, yielding more Go Timeouts, is entirely in line with the overall observation in the PD literature that dopaminergic therapy improves inhibition of inappropriate motor responses in PD.

It is now understood that dopaminergic treatment in PD leads to improvements in some aspects of cognition, but impairments in others (Cools et al., 2001; Rowe et al., 2008; MacDonald and Monchi, 2011; MacDonald et al., 2011). These complex cognitive effects are explained by differences in dopaminergic levels at baseline across different brain regions in PD. According to this view, dopaminergic therapy is titrated to a dose needed to replenish the dopamine-deficient DS and improve movement symptoms in PD. Dopaminergic therapy distributes in a non-targeted fashion, however, overdosing regions such as the VS and medial prefrontal regions that are at baseline dopamine-replete, innervated by the relatively-spared VTA (Gotham et al., 1986, 1988; Swainson et al., 2000; Cools et al., 2001; Cools, 2006; Vaillancourt et al., 2013). As a result, DS-mediated cognitive functions such as selective attention (Baunez and Robbins, 1999; MacDonald et al., 2011; de Manzano et al., 2013), decision-making (Balleine et al., 2007; MacDonald et al., 2011; Hiebert et al., 2014b), response inhibition (Zandbelt and Vink, 2010; MacDonald and Monchi, 2011; Wylie et al., 2012), and overriding pre-potent and automatic responses to enact more considered and accurate responding (Ali et al., 2009; MacDonald et al., 2011; MacDonald A. A. et al., 2014; Robertson et al., 2015) show improvements with the addition of dopaminergic treatment. This is entirely in line with our findings here in the Go/No-go task. In contrast, cognitive functions mediated by brain regions receiving dopamine from VTA such as reward processing, feedback learning (Swainson et al., 2000; Cools et al., 2001, 2007; MacDonald and Monchi, 2011; MacDonald et al., 2013b; Vaillancourt et al., 2013; Vo et al., 2014, 2017; Gallant et al., 2016), motivation (Humphries and Prescott, 2010; Simões-Franklin et al., 2010; MacDonald and Monchi, 2011), and orienting to stimuli (Zink et al., 2003; Jensen et al., 2007; MacDonald and Monchi, 2011; Esslinger et al., 2013; Anderson et al., 2016) are impaired.

Our finding in the Go/No-go task along with the results of the meta-analysis conducted by Manza et al. (2017), are compatible with *reduced* motor impulsivity due to dopaminergic therapy in PD. These effects co-exist with the recognition of ICDs arising with dopaminergic treatment (Pontone et al., 2006; Weintraub et al., 2014), reflecting *enhanced* cognitive/motivational impulsivity in PD. Though presenting opposite effects of dopaminergic therapy on cognition, these patterns are understood through the same framework provided above. DS has been implicated in *limiting* motor impulsivity by ensuring more considered and less habitual responding (Hood et al., 2007; Cools et al., 2010; Djamshidian et al., 2011; MacDonald et al., 2011; Ness and Beste, 2013; Robertson et al., 2015). In contrast, VTA-innervated brain regions such as VS and orbitofrontal cortex mediate motivation and reward processing (Balleine et al., 2007; Rowe et al., 2008; Drijgers et al., 2012). In PD, dopaminergic therapy normalizes DS dopamine deficiency and therefore predictably improves the ability to make deliberate and less impulsive responses as we see here (Balleine et al., 2007; Rowe et al., 2008; Drijgers et al., 2012). Conversely, treatment with dopaminergic agents overdoses VS and other VTA-innervated brain areas, dysregulating motivation and impairing reward processing, leading to ICDs. Our findings and the literature linking ICDs to dopaminergic therapy are easily reconciled, understanding that impulsivity is a multifaceted concept, with its various forms mediated by distinct brain regions that are differentially dopamine-depleted in PD and hence dissimilarly affected by dopaminergic therapy.

## Conclusion

Overall, we provide support for the role of dopaminergic therapy in decreasing motor impulsivity in PD. Our findings illustrate the importance of recognizing impulsivity as a multi-faceted construct. These findings enhance our understanding of the effect of dopaminergic therapy on cognition in PD. This knowledge will ultimately inform clinical decisions regarding dosing of dopaminergic therapy in PD, taking into account different cognitive as well as motor symptoms.

## Author Contributions

XQY, BL, KNS and PAM designed the experiment and edited the manuscript. XQY and BL conducted the experiment. XQY and PAM performed the analysis. XQY, BL and PAM wrote the manuscript.

## Conflict of Interest Statement

The authors declare that the research was conducted in the absence of any commercial or financial relationships that could be construed as a potential conflict of interest.
