# Supplementary material for: Dopaminergic Therapy Increases Go Timeouts in the Go/No-Go Task in Patients with Parkinson’s Disease
Source: Front Hum Neurosci. 2018 Jan 4;11:642. doi: 10.3389/fnhum.2017.00642 (PMC5758505; doi:10.3389/fnhum.2017.00642)
Supplement: Supplementary file 1 [file Table_1.docx]

Supplementary Material

Dopaminergic Therapy Increases Go Timeouts in the Go/No-go Task in Patients with Parkinson’s Disease

Xue Qing Yang, Brian Lauzon, Ken N. Seergobin, Penny A. MacDonald^*^

*** Correspondence:** Penny A. MacDonald: penny.macdonald@gmail.com

# Supplementary Figures and Tables

**Supplementary Table 1.** Demographic and cognitive measures for PD patients. Data are listed for all participants who were included in analyses. All values are in units of the respective questionnaire or task scale. N: number of participants; Education (years): number of years of secondary and post-secondary education; PD duration (years): number of years since PD diagnosis; LED (mg): Levodopa Equivalent Dose; NFOG: New Freezing of Gait Questionnaire/28; BIS: Barratt Impulsiveness Scale/120; SSS: Sensation-Seeking Scale/40; QUIP-RS ICD: Questionnaire for Impulsive-Compulsive Disorders in Parkinson’s disease Rating Scale – Impulse-Control Disorders/64; QUIP-RS Total: Questionnaire for Impulsive-Compulsive Disorders in Parkinson’s disease Rating Scale – Total score/112; MoCA: Montreal Cognitive Assessment/30; ANART: American National Adult Reading Test/135.6; COWAT FAS (number of words): Controlled Oral Word Association Test FAS Task; COWAT Animal (number of words): COWAT Animal Task; UPDRS: Motor Subscale Score of the Unified PD Rating Scale/56, listed for ON and OFF medication.

| Participant # | Age | Sex | Education | Handedness | PD Duration | LED | NFOG | BIS | SSS | QUIP-RS-ICD | QUIP-RS Total | MoCA | ANART | COWAT FAS | COWAT Animal | UPDRS OFF | UPDRS ON |
| --- | --- | --- | --- | --- | --- | --- | --- | --- | --- | --- | --- | --- | --- | --- | --- | --- | --- |
| 1 | 49 | Female | 13 | Right | 4 | 800.04 | 18 | 62 | 9 | 35 | 52 | 26 | 119.04 | 16.66667 | 26 | 10 | 5.5 |
| 2 | 66 | Male | 16 | Right | 1 | 600 | 7 | 45 | 17 | 31 | 57 | 28 | 125.48 | 16.33333 | 21 | 18 | 14 |
| 3 | 78 | Male | 15 | Right | 2 | 300 | 0 | 50 | 14 | 16 | 37 | 27 | 129.16 | 10.33333 | 10 | 23 | 17.5 |
| 4 | 61 | Male | 16 | Right | 4 | 568 | 0 | 48 | 21 | 7 | 11 | 30 | 124.56 | 17.66667 | 25 | 15.5 | 10.5 |
| 7 | 75 | Female | 12 | Right | 6 | 400 | 15 | 52 | 8 | 14 | 28 | 26 | 128.24 | 16 | 16 | 16.5 | 17 |
| 8 | 69 | Male | 18 | Right | 5 | 850.25 | 0 | 65 | 9 | 7 | 17 | 28 | 131.92 | 23 | 26 | 25 | 21.5 |
| 9 | 77 | Female | 16 | Right | 1 | 300 | 9 | 65 | 8 | 8 | 18 | 25 | 119.04 | 14 | 14 | 23.5 | 19.5 |
| 11 | 71 | Male | 15 | Right | 1 | 550 | 0 | 48 | 13 | 3 | 8 | 29 | 123.64 | 8.666667 | 19 | 20.5 | 18.5 |
| 12 | 75 | Male | 17 | Right | 3 | 798 | 10 | 65 | 14 | 19 | 40 | 24 | 126.4 | 14 | 19 | 23 | 19.5 |
| 14 | 61 | Male | 13 | Right | 3 | 650 | 9 | 59 | 15 | 10 | 15 | 27 | 117.2 | 7.333333 | 16 | 20.5 | 14 |
| 15 | 63 | Male | 15 | Right | 2 | 600 | 0 | 69 | 23 | 23 | 42 | 25 | 124.56 | 8.333333 | 10 | 24.5 | 19.5 |
| 16 | 71 | Male | 17 | Right | 2 | 400 | 0 | 70 | 16 | 22 | 38 | 25 | 125.48 | 20.66667 | 19 | 21 | 17 |
| 18 | 65 | Female | 13 | Left | 7 | 601.5 | 10 | 75 | 5 | 12 | 32 | 27 | 119.96 | 21.66667 | 30 | 15 | 13 |
| 19 | 44 | Male | 25 | Right | 11 | 1150.02 | 22 | 51 | 12 | 9 | 21 | 27 | 124.56 | 25 | 34 | 17.5 | 12.5 |
| 20 | 71 | Male | 12 | Right | 3 | 700 | 0 | 67 | 7 | 11 | 14 | 29 | 118.12 | 9 | 17 | 26.5 | 18.5 |
| 21 | 62 | Female | 18 | Right | 5 | 600.75 | 14 | 62 | 12 | 17 | 25 | 30 | 131.92 | 20.66667 | 32 | 22.5 | 19 |
| 22 | 68 | Female | 15 | Right | 26 | 949.25 | 18 | 63 | 7 | 11 | 23 | 29 | 131 | 19 | 25 | 41.5 | 39 |
| 23 | 58 | Female | 13 | Right | 11 | 851.5 | 12 | 47 | 9 | 10 | 30 | 28 | 116.28 | 18.33333 | 24 | 25.5 | 20.5 |
| 24 | 68 | Female | 13 | Right | 2 | 633.5 | 4 | 62 | 8 | 5 | 6 | 27 | 105.24 | 9.333333 | 10 | 27 | 20.5 |
| 25 | 72 | Female | 15 | Right | 12 | 1148.75 | 20 | 71 | 6 | 2 | 11 | 27 | 115.36 | 12.66667 | 22 | 19.5 | 12.5 |
| 26 | 83 | Female | 20 | Right | 3 | 300 | 0 | 57 | 10 | 4 | 4 | 30 | 125.48 | 17 | 24 | 21.5 | 14 |
| 27 | 62 | Female | 17 | Left | 1 | 33.5 | 5 | 50 | 12 | 11 | 19 | 27 | 117.2 | 20 | 28 | 22.5 | 20 |
